# 

**Published:** 1890-07

**Authors:** 


### NOTICE.

The seventh semi-annual meeting of the Ohio State Veterinary Medical Association will be held in the council chambers of Dayton, Ohio, on July 16th, 1890, at 8 o'clock P. M.

Papers will be read by Drs. G. W. Butler, S. R. T. Foward, W. P. F. Gribble and others.

Veterinarians from other state associations are cordially invited to attend.

W. J. Torrance, V. S., *Secretary.*

### BOOKS AND PAMPHLETS RECEIVED.

Thiermedizinische Vortrage. Bd. 1, Heft 11–21. Die neuren Antipyretica. von J. Tereg. Leipzig, 1890.

Annales de la Sociedad Cientifica Argentina. Tom XXIX. Ent. 2–3.

Journal of the Bombay Natu-al History Society. Vol. V, No. 1.

Annales et Bulletin de la Societé de Médecine de Gand.

Bulletin de L'Academie Royale de Médecine de Belgique.

Abhandlungen von naturwissenschaftlichen Vereine zu Bremen. 1890.

Societe Linneenne du Nord de la France.

Bulletin de la Societe Vaudoise des Sciences Naturelles. Vol. XXV, No. 100. Lausanne.

Mittheilungen des Naturwissenschaftlichen Vereines fur Steiermark. Gratz.

Jenaische Zeitschrift fur Naturwissenschaft herausgegeben von der medizinisch-naturwissenschaftlichen Gesellschaft zu Jena.

Journal of Cincinnati Society of Natural History. Vol. XIII, No. 1.

Report of the Council of the Zoological Society of London for the year 1889

Annual Report of the Zoological Society of Cincinnati, 1890.

Bollettino Scientifico. Anno XIII, No. 1. Pavia.

Der Zoologische Garten. Edited by Dr. F. C. Noll. Frankfurt-a-M.

Ornis. Jahr VI, Heft I. Vienna.

Le Naturaliste Canadiene. Cape Rouge.

Ottawa Naturalist.

Johns Hopkins University Circulars.

Bulletin No. 65, New Jersey Agricultural Experiment Station. New Brunswick, January 31, 1890.

Encyclopédie d'Hygiène et de Médecine Publique. Epizooties (Maladies des Animaux transmissibles a l'homme). By MM. Nocard and Leclainche. Paris, 1890. An interesting and valuable book of reference for the scientist and general information for the lay reader.

Rabies is treated in regard to its nature, the species susceptible to contract it, the clinical symptoms, lesions, etiology, pathogeny and prophylaxy, and sanitary police, in the most satisfactory manner that we have ever found. The article on glanders should be translated for students' review papers for examinations and for public information. Articles on Anthrax, Aphthous

Fever, Tuberculosis, Trichinosis, Actinomycosis and Echinococci are concisely arranged for reference.

Connecticut State Board of Health. Twelfth Annual Report, for 1889. New Haven, 1890.

Report of the Secretary of Agriculture, 1889. Washington, 1889.

The reports of the entomologist and botanist are of direct interest to veterinarians.

Massachusetts Agricultural College. Special Bulletin, May, 1890. Commercial manures. Bulletin No. 9. Soil tests with fertilizers.

Wallace's Year Book. Trotting and Pacing, 1889. Containing full summaries of all the year in which any heat was trotted or paced in 2.40 or less, carefully compared with and corrected from official reports of the National Trotting Association and the American Trotting Association. J. H. Wallace, New York, 1890.

Pennsylvania State Board of Health. Circulars for the Guidance of Heads of Families. Consumption, School Hygiene, Typhoid Fever, Contagious and Infectious Diseases, Scarlet Fever.

